# Retraction: FAM172A controls endoplasmic reticulum (ER) stress related to NF-κB signaling pathway in hepatocellular carcinoma

**DOI:** 10.1039/d5ra90015h

**Published:** 2025-02-19

**Authors:** Wenfeng Shen, Zhiqiang Feng, Ping Wang, Jinqian Zhang

**Affiliations:** a Department of Ultrasound, The Affiliated Hospital of Inner Mongolia Medical University Hohhot 010059 China; b Department of Hepatobiliary Surgery, Air Force General Hospital Beijing 100142 China fengzq1151@163.com +86-10-66928312 +86-10-66928312; c Department of Nuclear Magnetic Resonance, Air Force General Hospital Beijing 100142 China; d Department of Laboratory Medicine, Guangdong Second Provincial General Hospital, Southern Medical University No. 466 Xingang Middle Road, Haizhu District Guangzhou 510317 Guangdong Province China jingwanghou@163.com +86-20-89168239 +86-20-89168239

## Abstract

Retraction of ‘FAM172A controls endoplasmic reticulum (ER) stress related to NF-κB signaling pathway in hepatocellular carcinoma’ by Wenfeng Shen *et al.*, *RSC Adv.*, 2017, **7**, 51870–51878, https://doi.org/10.1039/C7RA09918E.

The Royal Society of Chemistry hereby wholly retracts this *RSC Advances* article due to concerns with the reliability of the data.

There are multiple pieces of western blot data that were first published in ref. 1, or were under review in ref. 2 at the same time as this *RSC Advances* article. There are no authors in common between this *RSC Advances* article and ref. 1 or ref. 2.

• The blots in Fig. 3j were first published as Fig. 5a in ref. 1. They were also published in Fig. 5a in ref. 2 and both articles were in peer review at the same time.

• The western blot data for Actin in Fig. 4a is a partial mirror image of the data for actin in Fig. 4c. Part of the western blot data for actin in Fig. 4a and c were first published as β-actin in Fig. 2b of ref. 1 and have also been published as β-actin in Fig. 2b of ref. 2.

• The western blot data for NF-κB in Fig. 4b was first published as Fig. 9a in ref. 3. It has been darkened and compressed.

• In Fig. 4c the blots for control and pcDNA6.2.RNAi-control for FAM172A are identical. The blots for pEGFP-c1 and pcDNA6.2.RNAi-control for cyclin A are identical. The blots for control, pEGFP-c1 and pcDNA6.2.RNAi-control for elF2α are identical.

• In Fig. 4c the left four blots for GRP78 are very similar to the blots for flotillin-1 in Fig. 2b of ref. 1, AdipoR1 in Fig. 2b of ref. 2 and TLR-4 in Fig. 2b of ref. 2.

• In Fig. 4c the left four blots for p-PERK are very similar to the blots for AdipoR1 in Fig. 2b of ref. 1 and AMPK in Fig. 2b of ref. 2.

• In Fig. 4c the left four blots for CHOP are very similar to the blots for AMPK and p-AMPK in Fig. 2b of ref. 1 and p-AMPK in Fig. 2b of ref. 2.

The cell images from Fig. 3b–e were first published in ref. 4.

The authors were asked to provide the raw data for this article, but did not respond. Given the significance of the concerns about the validity of the data, and the lack of raw data, the findings presented in this paper are not reliable.

The authors did not respond to any correspondence regarding the retraction of this article.

Signed: Laura Fisher, Executive Editor, *RSC Advances*

Date: 28th January 2025

**References**

1 L. Yuan, F. Shan and J. Chen, Lipid raft-mediated miR-3908 inhibition of migration of breast cancer cell line MCF-7 by regulating the interactions between AdipoR1 and Flotillin-1, *World J. Surg. Oncol.*, 2017, **15**, 69, DOI: 10.1186/s12957-017-1120-9

2 J. Liu, G. Li, C. Chen, D. Chen and Q. Zhou, MiR-6835 promoted LPS-induced inflammation of HUVECs associated with the interaction between TLR-4 and AdipoR1 in lipid rafts, *PLoS One*, 2017, **12**, e0188604, DOI: 10.1371/journal.pone.0188604

3 L. Li, J. Zhang, Y. Yang, Q. Wang, L. Gao, Y. Yang, T. Chang, X. Zhang, G. Xiang, Y. Cao, Z. Shi, M. Zhao and G. Gao, Single-wall carbon nanohorns inhibited activation of microglia induced by lipopolysaccharide through blocking of Sirt3, *Nanoscale Res. Lett.*, 2013, **8**, 100, DOI: 10.1186/1556-276x-8-100, (retraction published 2023, L. Li, *et al.*, *Discover Nano*, 2023, **18**, 130, DOI: 10.1186/s11671-023-03908-3).

4 K. Qian, J. Zhang, J. Lu, W. Liu, X. Yao, Q. Chen, S. Lu, G. Xiang and H. Liu, FAM172A modulates apoptosis and proliferation of colon cancer cells via STAT1 binding to its promoter, *Oncol. Rep.*, 2016, **35**, 1273–1290, (retraction published 2023, K. Qian, *et al.*, *Oncology Reports*, 2023, **49**, 107, DOI: 10.3892/or.2023.8545).

